# Determination of the optimal tubulin isotype target as a method for the development of individualized cancer chemotherapy

**DOI:** 10.1186/1742-4682-10-29

**Published:** 2013-05-01

**Authors:** Siamak Ravanbakhsh, Melissa Gajewski, Russell Greiner, Jack A Tuszynski

**Affiliations:** 1 Department of Computing Science, University of Alberta, Edmonton, AB, T6G 2E8, Canada; 2 Department of Oncology, University of Alberta, Edmonton, AB T6G 1Z2, Canada; 3 Department of Physics, University of Alberta, Edmonton, AB T6G 2E1, Canada; 4 Alberta Innovates Centre for Machine Learning, Edmonton, AB, T6G 2E8, Canada

**Keywords:** Bioinformatics, Drug design, Cancer, Tubulin, Side effects, Optimization

## Abstract

**Background:**

As microtubules are essential for cell growth and division, its constituent protein β-tubulin has been a popular target for various treatments, including cancer chemotherapy. There are several isotypes of human β-tubulin and each type of cell expresses its characteristic distribution of these isotypes. Moreover, each tubulin-binding drug has its own distribution of binding affinities over the various isotypes, which further complicates identifying the optimal drug selection. An ideal drug would preferentially bind only the tubulin isotypes expressed abundantly by the cancer cells, but not those in the healthy cells. Unfortunately, as the distributions of the tubulin isotypes in cancer cells overlap with those of healthy cells, this ideal scenario is clearly not possible. We can, however, seek a drug that interferes *significantly* with the isotype distribution of the cancer cell, but has only *minor* interactions with those of the healthy cells.

**Methods:**

We describe a quantitative methodology for identifying this *optimal tubulin isotype profile* for an ideal cancer drug, given the isotype distribution of a specific cancer type, as well as the isotype distributions in various healthy tissues, and the physiological importance of each such tissue.

**Results:**

We report the optimal isotype profiles for different types of cancer with various routes of delivery.

**Conclusions:**

Our algorithm, which defines the best profile for each type of cancer (given the drug delivery route and some specified patient characteristics), will help to personalize the design of pharmaceuticals for individual patients. This paper is an attempt to explicitly consider the effects of the tubulin isotype distributions in both cancer and normal cell types, for rational chemotherapy design aimed at optimizing the drug’s efficacy with minimal side effects.

## Background

Microtubule-targeting anti-mitotic agents are among the most successful drugs used for cancer treatment [1]. Unfortunately, since microtubules are essential for healthy as well as cancer cells, these drugs have many serious side effects [2]. However the existence of tubulin in multiple isotypic forms [3] presents an opportunity to design drugs that target primarily the isotypes expressed in cancer cells, but not the isotypes expressed in healthy cells, thereby suggesting a drug that is effective against cancer, with minimal side effects. This possibility has not been explored, nor has this line of attack been formalized, until now. This paper addresses the task of quantitatively characterizing the ideal binding affinity profile for such a drug, in a way that can be used for any family of existing or future tubulin-binding agents.

**Figure 1:**
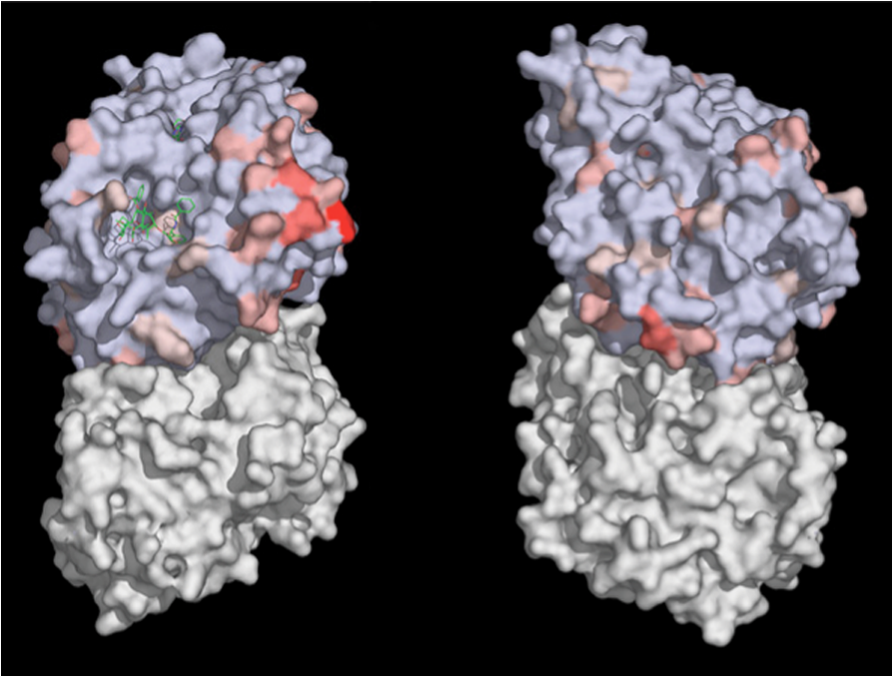
**Surface map of conserved residues in all human β-tubulin isotypes. **A solvent accessible surface was drawn onto the α/β tubulin heterodimer obtained by Löwe et al. [18]. An alignment of all human β-tubulin isotypes provided a score of overall homology at each amino acid position from 0–100%. This figure shows an α/β tubulin heterodimer, with the α-tubulin surface colored white and the β-tubulin surface colored blue and red. Individual surface residues within the β-tubulin monomer were colored to indicate the overall degree of homology ranging from light blue for 100% to bright red for 0–9%, with shades of red from brighter to paler illustrating those residues that are 10–39%, 40–79% and 70–99% homologous respectively. Two representations illustrate the paclitaxel bound interior of the microtubule and a 90° rotation about the y-axis to show the exterior of the microtubule. Figure reproduced from Huzil et al. [7].

Microtubules, which are cylindrical polymers composed of α/β-tubulin heterodimers, are involved in a wide range of cellular processes, such as the maintenance of cellular morphology and the active transport of cellular components throughout the cytoplasm [4,5]. An essential role for microtubules is the formation of the mitotic spindle, which produces the mechanical force required to separate the chromosomes [6]. A failure within this mitotic spindle apparatus leads to mitotic arrest and eventually apoptosis, resulting in cell death – which is desirable for cancer cells, but not for healthy tissues. As such, microtubules have become the target for a large number of anti-mitotic agents that act by either promoting or inhibiting microtubule polymerization by binding at specific sites on the exposed surface of α/β-tubulin heterodimers. Although there are multiple distinct binding sites on a tubulin heterodimer, β-tubulin is the main binding partner for all major microtubule-targeting drug families; hence we will focus on β-tubulin exclusively [7]. There are also several binding sites on β-tubulin; Figure 1 shows their location and residue differences between isotypes below. All microtubules inhibitors, including vinblastine, suppress microtubule dynamics [1] However, recent studies have found the role of microtubule dynamics in mitosis is a casual link [8] and microtubule inhibitors also act by multiple mechanisms like microtubule detachment (vinblastine, colchicine), or by hyperstabilizing microtubule organizing centers (paclitaxel) [9]. Some anti-tubulin drugs (e.g. vinca alkaloids) are especially effective since a few drug molecules can bind to the end of a microtubule and “poison” it by freezing its dynamic behavior – a behavior that is critical for microtubule function, especially in mitosis [10]. Our model exploits the fact that the binding affinity, measurably depends on the tubulin isotype involved.

Since essentially every cell in our bodies contains microtubules [6], drugs that affect microtubules have the potential to seriously disrupt healthy tissues, causing adverse side effects. The challenge is to design a drug that affects primarily cancer cells, while not significantly affecting healthy cells. Fortunately, this may be possible: tubulin protein (both α-tubulin and β-tubulin) is encoded by multiple genes. These isotypes are evolutionarily conserved, and may be functionally distinct [11]. Each tissue in the human body has its own characteristic distribution of these isotypes, and moreover, the expression levels of tubulin isotypes in cancer cells can differ from those in the originating normal tissues [12-14]. In fact, differences in the distribution of the βIII isotype in tumor samples have been correlated with patient treatment outcomes [15].

**Figure 2:**
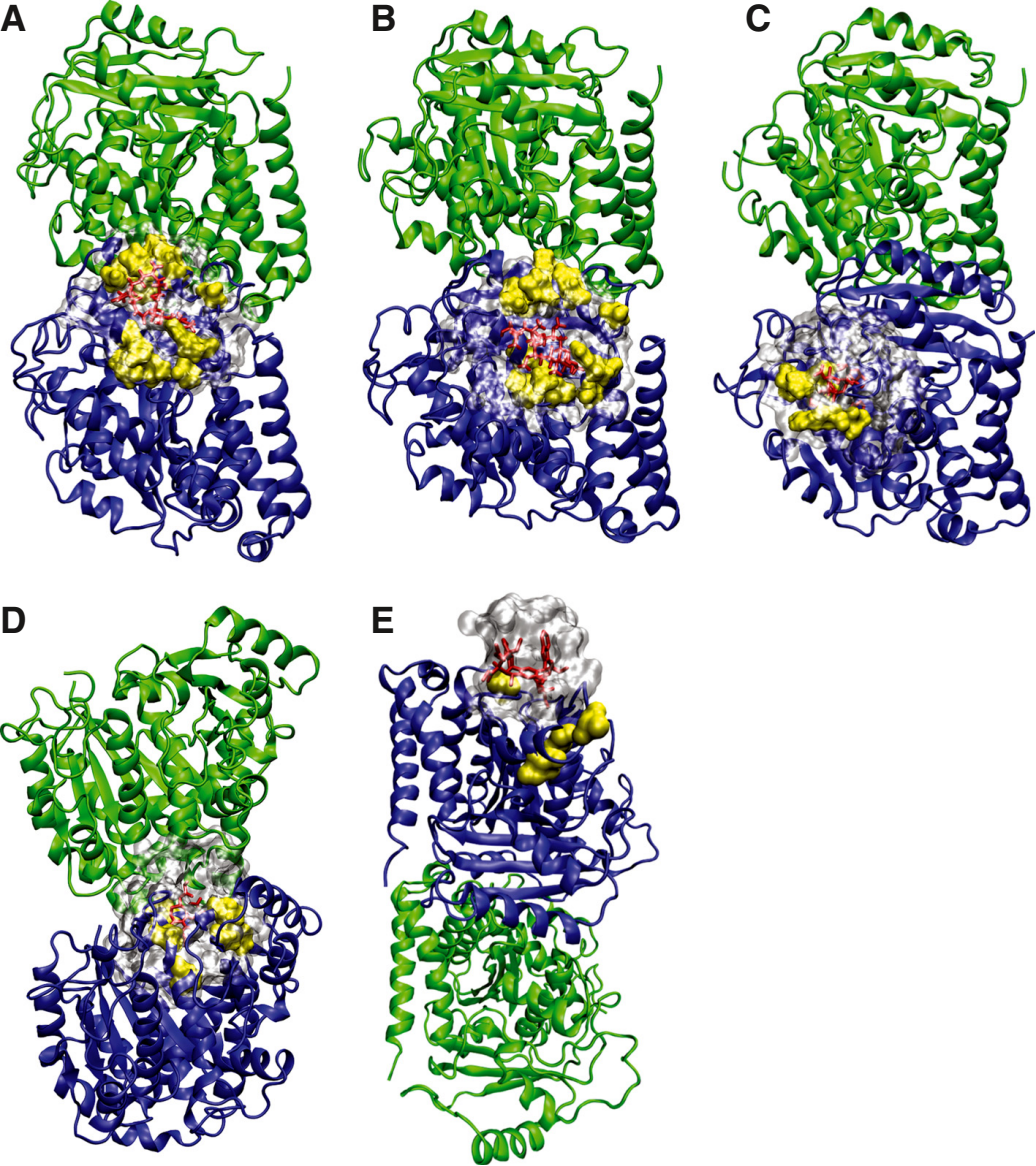
**Morphological differences in the drug binding pockets of β-tubulin isotype.** Visualization of the morphological differences in the binding pockets of β-tubulin isotypes for five selected drugs: (**A**) laulimalide, (**B**) peloruside A, (**C**) paclitaxel, (**D**) colchicine, and (**E**) vinblastine. α-Tubulin is shown in green, β-tubulin is shown in blue, the drug is shown in red, the drug binding pocket is shown in grey, and residue differences are shown in yellow. The paclitaxel complex is based on PDB:1JFF, the colchicine complex is based on PDB:1SA0, and the vinblastine complex is based on PDB:1Z2B.

More recent work on the significance of specific tubulin isotypes *vis-a-vis* chemotherapy agents supports those earlier studies, especially in regard to tubulin βIII and βV [16,17]. Importantly, the various β-tubulin isotypes differ in both the geometrical and biochemical properties of their respective drug binding sites, suggesting the design of anti-mitotic drug compounds with different affinities for different tubulin isotypes, allowing them to target a specific β-tubulin isotype over the others (see Figure 2) [7,18]. Although the numbers of residues that differ in each of the key binding sites are not great (see Tables 1 and 2 as well as Figure 1 for specific comparisons in several investigated examples), the overall effect on the binding affinity may be substantial, due to a simultaneous distal rearrangement of the protein structure (see Figure 2). Earlier calculations for specific families of drug compounds confirm these predictions [19,20]. Moreover, work is underway to quantify how these, sometimes minor, structural differences in the target translate into major changes in the binding affinity that result in the preferences for ligands to bind to specific tubulin isotypes. As this aspect is still under development for various tubulin-binding ligands, only fragmentary data are available. For example, Rowinsky et al. [2] measured the affinity of three vinca alkaloids (vinblastine, vincristine and vinorelbine) to brain tubulin isotypes (primarily βII and βIII plus a mixture of βI & βIV). Other studies examined the affinity of nocodazole [21] and colchicine [22] to tubulin isotypes, even though these drugs are not being used in cancer chemotherapy. Table 3 summarizes calculations, performed elsewhere [23], on two novel chemotherapy agents: peloruside A (PELA) and laulimalide (LAU) with respect to the β-tubulin isotypes, which illustrate how different isotypes of tubulin have different affinities for the same drugs. Further examples involving derivatives and analogues of these compounds [19] all indicate binding specificity and selectivity for these families of compounds. Similar conclusions have been recently reached for numerous colchicine derivatives [20]. This suggests it may be feasible to select chemotherapy compounds on the basis of their affinities for tubulin isotypes, which is the basis of the method we propose in this paper.

**Table 1:** Residue changes between β-tubulin isotypes in the binding sites for well-known drugs


**Isotype**	**Residue changes in the paclitaxel binding site**					
βIII				S275A		
βVI	V23M	S25G	D26E	S275A	R276Q	
**Isotype**	**Residue changes in the colchicine binding site**					
βIII		C239S			A315T	T351V
βV		C239S			A315T	T351V
βVI	V236I	C239S			A315T	T351V
**Isotype**	**Residue changes in the vinblastine binding site**					
βIII				T218A		

Residue changes between tubulin isotypes compared to βI tubulin with respect to the interactions with three well-known spindle poisons: paclitaxel, colchicine and vinblastine. Only residue differences in the respective binding sites are shown.

**Table 2:** Residue changes between β-tubulin isotypes in the binding site for peloruside A and laulimalide


**Isotype**	**Residue changes in the peloruside/laulimalide binding site**					
βIIa	C200S	V292M	A295S			
βIIb		V292M	A295S			
βIII		V292M				
βIVa		V292M				
βIVb		V292M				
βV		V292M	K296R			
βVI		V292M	K296R	M298T		
**Isotype**	**Residue changes in the peloruside/laulimalide binding site**					
βIII					N331A	V332I
βIVa					N331S	
βV					N331A	V332I
βVI	P304L	H306R	M329L		N331S	
**Isotype**	**Residue changes in the peloruside/laulimalide binding site**					
βIII	N334S					
βIVa	N334S					
βV	N334S					
βVI	N334T	K335R	Y339C			

Residue changes between β-tubulin isotypes with respect to the interactions with two novel spindle poisons, peloruside A and laulimalide, divided into three distinct regions that contribute to the interactions between the protein and the ligands.

**Table 3:** Binding energy differences for peloruside A and laulimalide as a function of the β-tubulin isotypes

	**βI**	**βIIa**	**βIIb**	**βIII**	**βIVa**	**βIVb**	**βV**	**βVI**
PELA	-22.6	-20.3	-10.6	-7.9	-18.2	-2.1	-22.0	0
LAU	0	0	-30.0	0	0	-21.9	-34.9	0

Binding energy differences in (kcal/mol) for peloruside A (PELA) and laulimalide (LAU) as a function of the β-tubulin isotypes. These values are based on molecular dynamics calculations [19].

There are many challenges with this framework; the Discussion section below summarizes many of these issues, and presents ways to address them.

## Methods

The ideal situation would obviously be to use a drug (a so-called “silver bullet”) that binds only to some specific tubulin isotype found exclusively in cancer cells. Unfortunately, there is no such isotype or any such drug. Instead, we will characterize the binding affinity profile for a hypothetical drug that has strong binding affinities for the β-tubulin isotypes that tend to be expressed in a given type of cancer, but relatively low binding affinities for the β-tubulin isotypes that tend to be expressed in healthy cells.

Our goal is to characterize the ideal binding affinity profile for a β-tubulin-targeting drug, given the expression levels of β-tubulin isotypes in healthy tissues and in cancer cells. To quantify the algorithm, we need to resolve four key issues:

A. Obtaining the expression levels of β-tubulin isotypes in various types of cells, both cancerous and healthy.

B. Quantifying the “damage” a drug will cause to a cell type, given both the binding affinities of the drug for the various β-tubulin isotypes, and the cell type’s β-tubulin isotype distribution. We define damage (used synonymously with injury) as the percentage of cells killed by the given dosage of the chemotherapeutic compounds.

C. Deciding on the relative importance of “protecting” each type of healthy tissue.

D. “Scoring” each proposed drug profile, in terms of its ability to both attack a given cancer type while protecting the important healthy tissues – we will use this scoring function to define which profile is optimal.

We consider 8 isotypes of human β-tubulin denoted as T = {βI, βIIa, βIIb, βIII, βIVa, βIVb, βV, βVI}. Leandro-García et al. [12] recently addressed issue A, by determining the expression levels of the mRNA for the corresponding genes {TUBB, TUBB2A, TUBB2B, TUBB3, TUBB4A, TUBB4B, TUBB6, TUBB1} in various healthy tissue and tumor samples, which is known to correlate with protein level [24], and so quantifies the importance of each isotype to each cell type. (Of course, our use here of mRNA, and not protein expression, means this is just an approximation; see the Discussion section.)

Each cell type’s isotype profile $q\left(c\right)\;=\;\left[q_{I}^{c},q_{\mathrm{IIa}}^{c},\ldots ,q_{\mathrm{VI}}^{c}\right]$ corresponds to these values. We view each profile as a probability tuple, as each entry is non-negative, and the 8 values in the tuple sum to 1.

Each (either already existing or proposed) drug *d* also has an associated profile of binding affinity values over these 8 isotypes, denoted as $r\left(d\right)\;=\;\left[r_{I}^{d},r_{\mathrm{IIa}}^{d},\ldots ,\;r_{\mathrm{VI}}^{d}\right]$, which quantifies the effect of drug *d* on the β-tubulin isotypes. Again, this is a probability 8-tuple, summing to 1. Each *r*_*i*_^*d*^ corresponds to how well the drug *d* binds to the *i*-th isotype, which is often written Δ*G*_*i*_(*d*) [23]. For a given type of cancer *c*, we want a drug *d* that is very damaging to *c*; we quantify this as a real value *D*(*r*(*d*), *q*(*c*)) that measures how much drug *d* damages cancer *c*. We define this using the simple dot product:

(1) $$D_{\text{dot}}\left(r,q\right)\;=\;r^{T}q\;=\;\sum_{i\in I}r_{i}q_{i}$$

While we focus on this linear model mainly for pedagogical reasons, we will see it also corresponds to log IC_50_; see Equation (5) in the Discussion section.

Our model deals with these drug molecules that bind only to β-tubulin (across various type of cells), but have no off-target interactions, i.e. no interactions with other proteins. However, these drug molecules have statistical preferences for different isotypes of tubulin, depending on the structure, that dictates the resultant affinity. Examples of colchicine derivatives, peloruside A, laulimalide, mentioned above, have been described in detail elsewhere [19,20].

To maximize the damage to the cancer cells *c*, we want a drug *d* so that *D*(*r*(*d*), *q*(*c*)) is as large as possible. At the same time we prefer drugs that do not damage the healthy tissues – that is, for each type of healthy tissue *t*_*h*_, we want the quantity *D*(*r*(*d*), *q*(*t*_*h*_)) to be *small*, to restrict the effect of the drug *d* on the isotypes expressed by *t*_*h*_. The third challenge listed above (issue C) is quantifying this tradeoff: how much should we protect the healthy cells, while still damaging the cancer cells. We address this by using a set of weighting values {*w*_*h*_}, where each *w*_*h*_ is used as a cutoff for the damage allowed to healthy tissue *t*_*h*_ – i.e., we will only consider a drug *d* if its distribution of tubulin isotype affinities, *r* = *r*(*d*), satisfies the constraint

(2) $$D\left(r,\;q\left(t_{h}\right)\right)\;\leq \;w_{h}$$

for each healthy tissue *t*_*h*_, we will eliminate any drug that violates this constraint for *any* healthy tissue *t*_*h*_.

Each *w*_*h*_ weight reflects the importance of the protection of tissue *t*_*h*_ to the patient’s survival (i.e., how much drug toxicity is tolerable), as well as the absorption of this drug into the cells of this type, which depends on the route of drug delivery and the binding affinities. In particular, we compute the weight *w*_*h*_ = *w*(*t*_*h*_; *c*, *a*, *p*) associated with healthy tissue *t*_*h*_ when dealing with cancer *c*, where the drug is delivered using route of administration *a* ∈{IV (intravenous infusion), local (intratumoral injection), oral, inhalant} to a person characterized by *p,* which includes gender *g* ∈ {male, female}, as well as other factors. (We include “inhalant” here, even though we realize there are currently no inhalant-based cancer chemotherapy treatments, even for lung cancer. However, there are preclinical trials involving aerosols of nanoparticles containing genotoxic agents [25]. Our analysis may suggest that anti-mitotic agents be tested for inhalant delivery for lung cancer and other cancers of the respiratory tract.) While the specific weights used here are somewhat arbitrary at this stage, note that our model allows these values to depend on the specific patient (for example dictated by co-morbidities and the health status) and the stage of the disease, as well as a doctor’s decision about the desired aggressiveness of the treatment regimen. Our approach is simple and robust enough to adapt to any selection of weight values.

**Figure 3:**
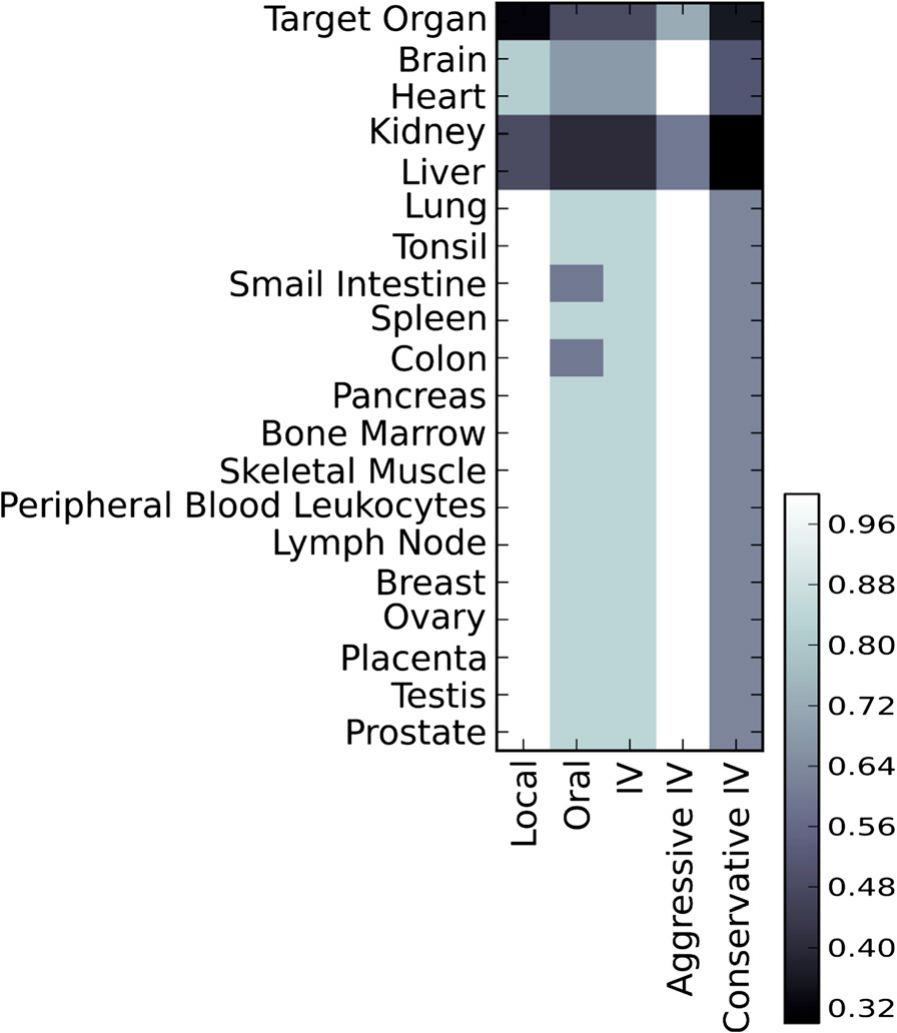
**Weighting values for tissues. **Weights used as cutoff values for the damage allowed to healthy tissues, for various combinations of tissues and routes of drug delivery. Our model rules out any potential drug that would cause too much damage to any health tissue. This maximum damage is based on a value *w*_*h *_= *w*(*t*_*h*_; *c*, *a, p*) for each healthy tissue *t*_*h*_, applied using administration “*a*” to a person characterized by “*p*”, who has cancer type “*c*”; see the constraint, Equation (4). This figure encodes these basic *w*_*h*_ values, for each the tissue *t*_*h *_(vertical axis) and administration “*a*” (horizontal). The bottom right shows the legend for the colors. (The text discusses how these weights are modified based on personal characteristic “*p*”).

Figure 3 shows the weighting values *w*_*h*_ = *w*(*t*_*h*_; *c*, *a*, *p*) for different combinations of route of administration *a* and tissue *t*_*h*_, as encoded by the color at each position. It does not show patient characteristics here, but note that it includes gender-specific organs only when considering patients of the associated gender – e.g., we will only show testis when dealing with male patients, and only show placenta when considering females. Otherwise this gender-specific organ is simply ignored in the constrained optimization. Again, these are simplified examples, intended to illustrate that the method can be further adapted to specific cases and situations.

The basic idea is to mildly protect every healthy organ present (which depends on the patient’s gender) for all methods except local delivery; in the case of local delivery, the tumor site (labeled “Target Organ” in Figure 3) is heavily protected, but the required protection for all other organs is weakened; the associated weight *w*_*h*_ is elevated by 20% over that baseline. The weights for brain and heart are relatively small values for all delivery routes, as they are vital organs and so must be further protected. Since drug compounds are metabolized in the liver, and the kidney accumulates foreign substances for excretion, these two organs will be more exposed to the drug and so they must receive even better protection (i.e., they are assigned smaller weights). The colon and small intestine are further protected in the case of oral delivery due to their direct exposure to the drug. Figure 3 does not show the weights for inhalant delivery as we consider it a “local” route of administration, with lung as its (additional) target organ. Again, the specific choice of values for the weights is somewhat arbitrary at this stage, due to the novelty of the method proposed. Once empirical data are available from tests, these numbers can be set accordingly. This versatility and robustness is a feature of our method.

However, our model explicates the need to protect specific organs from serious adverse side effects; this is consistent with the known side-effects of anti-tubulin drugs used in cancer chemotherapy [26]. One of the main toxicities of the vinca alkaloids is constipation, due to damage to the colon. (Other problems are neuropathy and neutropenia, due to damage to nerve and blood cells, respectively – cells not included in the current analysis due to the lack of quantitative data. Hopefully, tubulin expression information for these and other cell types will become available in the near future.) Similarly, both paclitaxel and docetaxel often lead to neurotoxicity and neutropenia (again, due to damage to nerve cells and blood cells, respectively).

Figure 3 also presents various patient-specific characteristics. For example, if a patient is in a weakened state of health, we might want to tighten all protection levels to 75%, meaning we require the drug to be uniformly “gentler” on every cell type, even if this means the drug is not as aggressive on the cancer; we call this approach “conservative”. Alternatively, a robust person might be able tolerate more drug toxicity; hence the “aggressive” personal characteristic where the weights are set to 150% of normal values. These patient-specific characteristics could be assessed by the doctor based on the patient’s medical history and current exam results. While the specific values of the weights chosen here are only for illustration purposes, note they can be adjusted for individual patients – this is a strength of our method.

The final challenge (issue D) is defining how to use this information – cancer type *c*, set of healthy tissues {*t*_*h*_}, weights {*w*_*h*_} (which recall depend on the cancer type *c*, route of administration, and patient characteristics), and profile function *q*(.) – to evaluate a particular profile. Here, we define the *optimal tubulin isotype profile* (OTIP) as

(3) $$\begin{array}{c} \text{OTIP}\left(c;\;q\left(.\right),\;\left\{w_{h}\right\}\right)\;=\;\text{arg}{\text{max}}_{r}\left\{D\left(r,q\left(c\right)\right)\right\},\\ \text{such}\;\text{that}\;\forall h:\;D\left(r,q\left(t_{h}\right)\right)\;\leq w_{h} \end{array}$$

where the index *h* ranges over the healthy tissues. That is, we want to inflict as much damage on the cancer cells, while insuring that the damage to each healthy tissue *t*_*h*_ is below the tissue-specific threshold *w*_*h*_.

If we are only interested in killing the cancer cells (i.e., if we only consider the first part of Equation (3)), then the OTIP for a tumor just puts all of the weight on only the single isotype with the highest expression in the cancer type *c* – i.e., argmax{*q*_*i*_ | *q*(*c*) = [*q*_I_, …, *q*_VI_]}. However, as we are constraining the optimization to cap the damage to the healthy cells (while also trying to maximize the damage to the cancer cells), we typically arrive at a different drug profile – one that often involves two or more isotypes that have only moderate affinity. Note finally that Equation (3) is easy to solve, as everything (both the constraints and the optimization formula) is linear [27-29].

## Results

**Figure 4:**
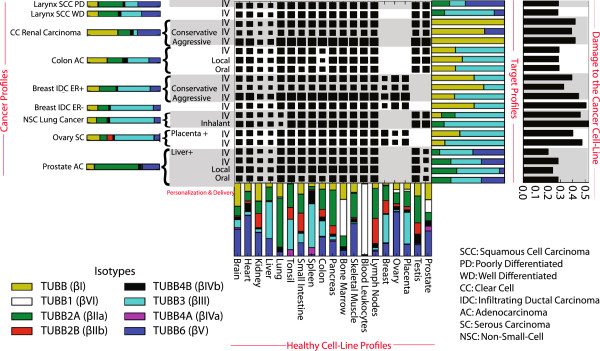
**Optimal tubulin isotype profiles. **Each row of the central matrix corresponds to a particular cancer-context *i *= (*c*, *a*, *p*) – that is, the type of cancer *c *(with the associated distribution *q*(*c*), shown by the horizontal bar on the left whose component regions are colored based on isotypes), how the drug is delivered *a*, as well as patient characteristics *p*, when relevant – e.g., we used a “+” symbol to indicate extra protection for a particular organ. Each column corresponds to a particular type of healthy tissue *t*_*h *_(with its name on the bottom, just below its associated profile *q*(*t*_*h*_)). The size of the box at position (*i*, *t*_*h*_) – for cancer-context *i *= (*c*, *a*, *p*) and healthy tissue *t*_*h *_– corresponds to the value of *w*_*h *_= *w*(*t*_*h*_; *c*, *a*, *p*), specifying the constraints on the damage to *t*_*h*_. (Larger squares correspond to larger *w*_*h *_values, which means the associated organ can tolerate more damage, and so requires less protection.) The right side shows the isotype distribution for the optimal drug, and the black bars (farthest right) show how much this optimal drug damages the associated cancer.

As an example of interpreting Figure 4, the first row deals with Larynx Squamous Cell Carcinoma, Poorly Differentiated (Larynx SCC PD). The adjacent colorful horizontal bars is the isotype profile for this tumor, followed by “IV”, indicating intravenous administration. The following row (in the matrix of squares) quantifies the protection level required for the various healthy tissues, for this type of cancer with this delivery: small boxes for liver and kidney to means that the drug is not permitted to cause too much damage to these organs; then larger-sized boxes for heart and brain, meaning these organs can tolerate more damage; and yet larger boxes for the remaining organs (from tonsil through lymph node, and testis to prostate), meaning these can take yet more damage. The entries for breast, ovary and placenta are blank (i.e., no constraints), for this male patient. The colorful bar to the right is the OTIP profile – i.e., the affinities of the drug that optimizes Equation (5): 25% βIIA, 21% βIII and 54% βV tubulin. The final black bar shows that this drug will be “0.29-damaging” for this cancer.

### Larynx squamous cell carcinoma

The top two rows in Figure 4 correspond to the cancers Larynx Squamous Cell Carcinoma Poorly Differentiated (Larynx SCC PD) and Larynx Squamous Cell Carcinoma Well Differentiated (Larynx SCC WD); the adjacent horizontal bars present the isotype profiles for these two tumors. We see slight differences, where Larynx SCC PD expresses more βIIa tubulin than Larynx SCC WD, but less βIII tubulin. Continuing left-to-right, the next entry is IV, indicating we are considering intravenous delivery of the drug. The following matrix of squares shows the protection weights, which quantify the protection for healthy tissues. In the PD row, we see that heart and brain have intermediate-sized boxes, but liver and kidney each have relatively small squares, which mean the drug is not permitted to cause too much damage to these organs. Then each tissue from tonsil through lymph node has a very large box, which means these organs are permitted to accept a relatively increased amount of damage. The entries for breast, ovary and placenta are blank (meaning no constraints), as we are considering a male patient. The second row in this matrix (Larynx SCC WD) is identical to Larynx SCC PD, as we are considering the same cancer site, and the same delivery method and same type of patient. To the right of the matrix is the OTIP profile – i.e., the profile of the drug that optimizes Equation (3). For Larynx SCC PD, the optimal drug’s affinity profile is 25% βIIA, 21% βIII and 54% βV tubulin; the subsequent black bars (on the far right) show that this drug will be “0.29-damaging” for this cancer. (The damage scale is a unit-less relative measure where higher values indicate the ideal drug profile will be more damaging to the cancer.) Notice the Larynx SCC WD row has the same three isotypes listed, but with very different proportions; in this case the optimal profile is 0.28-damaging for this cancer.

Among the drugs used for cancer of the larynx [26] (including cisplatin, 5-fluorouracil, carboplatin, and gemcitabine), both paclitaxel and docetaxel work by binding tubulin, notably with strong binding to βIIa and βIIb tubulin, then with less strength to βIII and βIV tubulin [30]. While our analysis suggests that these drugs might not be ideal, they are nevertheless affecting two of the four relevant isotypes.

### Clear cell renal carcinoma

The next three rows are all Clear Cell (CC) Renal Carcinoma, but with different delivery methods: normal IV (row 3), Conservative-IV (row 4) and Aggressive-IV (row 5). Here, we see the difference caused by the delivery method, as each Conservative-IV square is slightly smaller than the normal-IV squares, which means the Conservative-IV drug is allowed to do much less damage to each of the healthy cells – i.e., in this case it is (relatively) more important to protect the healthy tissue than to damage the cancer. So while the optimal target drug profile for IV administration is simply pure βI tubulin affinity, the Conservative-IV version of the drug profile “dilutes” this with 27% βV tubulin. As expected, we see that the Conservative-IV drug application method is slightly less damaging to this cancer (0.39-damaging) than the normal IV drug administration (0.42-damaging). The Aggressive-IV version has license to do even more damage to healthy cells than does IV. However, its ideal drug targets the same pure βI tubulin that the IV method prefers, and so this too is 0.42-damaging. Of course, we are not claiming that one should change policies on the basis of such a small change. We will later see more significant differences for other pairs of possible treatments.

Unfortunately cytotoxic drugs, such as tubulin-targeting agents, have been of little use in renal cancer therapy, with response rates below 15%. Of those agents, vinca alkaloids (which primarily target βII tubulin [31]) have been extensively used. It appears that the failure of these drugs is mainly due to the lack of efficacy and not due to side effects. Consequently, our recommendation would be to develop drugs with a strong affinity for βI tubulin, as well as some interactions with βV tubulin.

### Colon adenocarcinoma

The next three lines also deal with one type of cancer but several different types of delivery – Colon Adenocarcinoma (Colon AC), with IV, local and oral delivery. The ideal drug profiles for these three cases all involve the same two isotypes (βI and βIII) as targets, but in different proportions, as they need to protect the healthy organs differently.

While there are currently no tubulin-binding agents included in the standard therapy for Colon Adenocarcinoma, our analysis suggests targeting βI and βIII.

### Breast infiltrating ductal carcinoma

The next four rows are all Breast Infiltrating Ductal Carcinomas (Breast IDC); the first three are ER + (with three different delivery methods) and then the fourth is ER - (IV delivery). Here, we see squares for breast, ovary and placenta, as this patient is female. We again see different ideal profiles for these four cases, involving various distributions of βI and βIII, with effectiveness ranging from 0.33-damaging to 0.45-damaging.

Standard chemotherapy for breast cancer, however, currently involves the use of paclitaxel or docetaxel by IV infusion, which bind most to βIIa, βIIb, then to βIII and βIV tubulin [30]. Derry et al. [30] stated that the effects of taxanes on microtubules are assessed indirectly via their influence on microtubule dynamics, which is correlated with their binding affinity for tubulin, although subtle issues of distal structural effects, transport and intermediate binding sites may cloud the issue somewhat. Here, our analysis suggests seeking a variant that instead binds mostly to βI and slightly to βIII.

### Non-small-cell lung cancer

The next two rows deal with Non-Small-Cell (NSC) Lung Cancer; here we consider Inhalant as well as IV delivery methods. The only difference is in how much we need to protect the lungs; this in turn leads to a fairly different optimal profile: while both involve βIIa and βIII, the IV delivery also involves the βI isotype, which does not appear when dealing with the inhalant delivery. Note that inhalant has a better “cancer damage rate”, suggesting this is a better drug delivery method for NSC Lung Cancer.

This is consistent with the standard chemotherapy for NSC Lung Cancer, currently involving the use of paclitaxel (which targets βIIa and βIII). Note that paclitaxel also targets βIIb and βIV; our analysis suggests trying a novel drug that does not involve these additional targets.

### Ovary serous carcinoma

The two Ovary Serous Carcinoma (Ovary SC) rows differ based on how much we need to protect the healthy ovary and placenta cells. Both involve targeting βI and βIII tubulin. As expected, the additional constraints reduce the “kill rate” to the cancer cells.

Docetaxel is currently used to treat ovarian cancer, and it is administered through IV infusion. In analyzing 11 ovarian cancer samples (7 untreated, and 4 paclitaxel-resistant), Kavallaris et al. [32] found the 4 paclitaxel-resistant samples showed an increase in the expression of tubulin isotypes βI (3.6-fold), βIII (4.4-fold) and βIV (7.6-fold). Our analysis using OTIP explicitly identified two of these isotypes as specific targets for chemotherapy which is intriguing, especially in view of the fact that paclitaxel exhibits the greatest affinity for βII and not βI. Consequently, it would be logical to attempt directing chemotherapy for this type of cancer towards a βI-targeting agent. Obviously, whenever possible in ovarian cancer, it would be best to remove the ovaries altogether rather than attempt to spare part of it. However, if the patient involved has inoperable ovarian cancer, our strategy should be useful in minimizing side effects. Additionally, side effects on nerves or bone marrow should also play a significant role in deciding on the best strategy for chemotherapy selection.

### Prostate adenocarcinoma

Finally, we consider three types of drug delivery routes (with a personalization) associated with Prostate Adenocarcinoma (Prostate AC); Figure 4 shows the resulting optimal profiles and kill-rates. The “optimal” profile for targets involves tubulin isotypes βIIa, βIII and βV. This may at first appear paradoxical since the profile of isotypes expressed in prostate adenocarcinoma, as shown in Figure 4, includes isotypes βI, βIIa, βIVb and βV but not βIII. Note, however, that our method attempts to simultaneously maximize damage to cancer cells and minimize damage to healthy tissue, possibly with specific protection to certain tissues of significance. Moreover, affinity for one isotype does not completely exclude other isotypes. Having said this, due to its systemic action, chemotherapy is not commonly used to treat prostate cancer.

## Discussion

The present paper proposes an *in silico* method of designing a personalized chemotherapy for several types of cancers, designed to maximize the drug’s damage to the cancer cells while simultaneously minimizing the adverse side effects to healthy tissues. Our immediate goal is to propose a novel methodology for identifying the most appropriate drug. This required resolving four key issues to define our OTIP optimization model; we can consider other variants for all four of them. This Discussion section summarizes some such variants, then lists some challenges with our framework, and finally, some further advantages and extensions.

### Issue A: Obtaining expression levels of β-tubulin isotypes in various types of cells

First, if we later get more accurate measurements of the β-tubulin isotype expression distributions for the various cancer types and/or the healthy tissues, we can just plug them into the profiles *q*(.). Second, as noted earlier, the numbers presented here are based on mRNA levels [12]; while they are generally correlated with protein levels, this is only an approximation.

### Issue B: Quantifying the “damage” a drug will cause to a cell type

In general, *D*(*r*(*d*), *q*(*c*)) measures the cytotoxicity of a drug *d* with tubulin-binding *r*(*d*), on a cell *c* with profile *q*(*c*). Our linear model (Equation (1)) is partially motivated by the

log IC_50_(*c*,*d*) for a given cell line c, which utilizes the binding free energy between each tubulin isotype *i* and the drug, *d,* written Δ*G*_i_(*d*):

(5) $$D\left(r\left(d\right),\;q\left(c\right)\right)\;\alpha \;-\;\frac{1}{\mathrm{RT}}{\text{logIC}}_{50}\left(c,d\right)\;=\;{\langle \Delta G\left(d\right)\rangle }^{c}\;=\;\sum_{i=1}^{8}q_{i}\left(c\right)\;\Delta G_{i}\left(d\right),$$

where *q*_*i*_(*c*) (often written *P*_*i*_^*c*^ ) continues to represent the relative abundance of a particular tubulin isotype *i* in the cell line *c*, *T* is the temperature in Kelvin and *R* = 8.31 J/(mol K) is the gas constant [23]. We use this very simple model to provide a framework for our methodology. Of course, it does not incorporate many other important issues, such as A or B or many of the other issues listed by Ganguly et al. [9].

Notice it is straightforward to modify our framework to consider yet other “damage” models – indeed, we formulated the task using the abstract *D*(*r*, *q*) notation, to make it easy to “plug in” appropriate formulas that relate the drug binding affinity profile *r* to the cell’s isotype profile *q*. It can be extended to incorporate known interactions with other proteins (e.g., paclitaxel’s known interactions with apoptosis effectors), provided these interactions have been properly modeled. We anticipate further researchers will be able to use their own *D*(*r*, *q*) functions, within our framework.

As one specific example, we can replace Equation (3) with the (negative of the) Kullback–Leibler divergence [33-35]

(4) $$D_{\mathrm{KL}}\;\left(r,\;q\right)\;=\;-\sum_{i\in T}r_{i}\;\text{log}\frac{r_{i}}{q_{i}},$$

which is always non-positive, and is 0 iff *r* = *q*. Here, if we ignore the constraints of avoiding too much damage to healthy cells, then the associated OTIP_KL_ optimization (using this *D*_KL_ rather than *D*_dot_) for a tumor will be identical to the tumor’s profile – i.e., the profile for the OTIP_KL_ drug *r* will match the profile of a cell type *c*. This is in contrast with the current OTIP algorithm (based on *D*_dot_(*r*, *q*) ), which selects the isotype with the highest expression level. This demonstrates how these two measures embody different assumptions about the best way to disrupt the functionality of the β-tubulin isotypes: disrupting the most abundant isotype versus disrupting all isotypes proportionally.

### Issue C: Deciding on the relative importance of “protecting” each type of healthy tissue

Similarly to the situation surrounding issue A, we may later be able to use more precise biological knowledge to improve the way we assign the {*w*_*h*_} weighting values – i.e., finding a better way to decide the relative importance of protecting each type of healthy tissue). This could be generic, for all patients, or it could be specific to a particular patient – i.e., this would be the basis of completely personalized medicine.

### Issue D: “Scoring” each proposed drug profile

Finally, Equation (3) is a constrained optimization task, which rejects a proposed drug if it violated any constraints. Alternatively, we could instead just consider a “total cumulative score”, which is the tumor damage minus the weighted sum of the damage to the various healthy cells – i.e., of the form

(6) $$\text{OTI}P_{\text{sum}}\left(c;q\left(.\right),\left\{v_{h}\right\}\right)\;=\;\text{arg}{\text{max}}_{r}\left\{D\left(r,\;q\left(c\right)\right)-\sum_{h}v_{h}D\left(r,\;q\left(c_{h}\right)\right)\right\},$$

which here is using weights *v*_*h*_ over the healthy cells, which differ from *w*_*h*_ in that it uses larger *v*_*h*_ values for cell types that need more protection (recall this would mean we *decrease w*_*h*_).

In all cases, it is an empirical question to determine which actual “setting” of these four issues is truly the best one – i.e., which leads to the best results, in terms of (average) mortality and morbidity of the patients.

We close this section by discussing ways to address several possible limitations of our framework, followed by some further advantages and extensions.

### General issues with targeted drugs

Some issues are problems inherent in any targeted drug (whether identified by OTIP or not.) For example, as cancer is a very complex disease with equally complex drug resistance mechanisms, any cancer chemotherapy drug, including the ones suggested here, can fail in part because a tumor is heterogeneous and can transform to a new lineage.

There are also many factors that complicate both the design and delivery of tumor-suppressing chemotherapy, such as the presence of efflux pumps, multi-drug resistance, p53 mutations, off-target interactions, angiogenic vasculature, mitotic slippage, heterogeneity of the tumor cells, their higher mutability, alterations in tubulin isotype expression over time and the potential to develop drug resistance [36,37]. These complications mean that producing an effecting drug (perhaps one that is OTIP-optimal) is not sufficient to guarantee effective treatment.

Another set of issues are implicit within the OTIP framework. Following standard practice, we implicitly assume a single (homogenous) type of cancer and deal only with a single time point. There are obvious extensions possible for handling known heterogeneity, by dealing with the distribution of cancer subtypes. One might first approximate this by assuming the damage that a drug with profile *r* causes a distribution of cancer cells is just a linear combination of the damage that *r* causes each individual subtype of cell – using a formula analogous to Equation (8). We also know that cancer can change over time. This may be because tubulin expression is altered by exposure to drugs, which is thought to involve miRNA [38]. We may also need to re-weight over time the tolerance of some of the healthy tissues, e.g., to deal with hepatotoxic side effects of drugs that challenge the liver. It is easy to address this: after exposure to some drugs, we could use a biopsy to determine the altered levels of tubulin expression in tumor cells, and then re-run OTIP on this new profile to recalculate the optimized drug composition.

### Estimating *r*(*d*) for drug *d*

This result of our OTIP analysis is a description of the β-tubulin isotype profile *r* of an ideal drug. A remaining challenge, of course, is then synthesizing a drug *d* with this profile – i.e., *r*(*d*) *= r*. A related goal is evaluating a given drug *d* – that is, computing (or at least estimating) *r*(*d*)*.*

The specific structural differences between isotypes means a given drug will bind differently to various isotypes. Table 3 shows two specific cases of tubulin-binding drugs, peloruside A (PELA) and laulimalide (LAU). Similar differences are expected to exist for other families of compounds, including vinca alkaloids, taxanes, colchicines, epothilones, etc., but to the best of our knowledge, no systematic analysis of these effects exists in the literature. In the absence of sufficient experimental data sets that show the binding rates of the various compounds to all tubulin isotypes, these differences between isotypes can nevertheless be estimated computationally. We can predict binding probabilities for each isotype using an Arrhenius-type formula, where the probability of drug *d* binding an isotype *i*, *r*_*i*_(*d*), is proportional to the exponential function of the binding free energy [23], Δ*G*_*i*_(*d*) – that is

(7) $$r_{i}\left(d\right)\;\sim \;\text{exp}\left(-\beta \Delta G_{i}\left(d\right)\right).$$

### Validation of the framework

While there is currently no way of evaluating the assumptions proposed in our paper, there are at least three possible ways to address this issue in the future: **(a)** Obtain the necessary experimental data to show that the assumptions are justified. Such data could include measuring the drug efficacy values for each tissue type and drug delivery route, assessing patient-to-patient variation of isotype distributions in different normal and cancer impacted tissues, and validating tissue-specific “allowed damage” cutoffs. This, however, requires a prolonged concerted effort in amassing information that may be fragmentary and scattered throughout the literature. **(b)** Alternatively, we could show that OTIP method is not overly sensitive to the various parameter choices. We are currently working on this aspect. As this is a linear model, small perturbations that retain feasibility will have small effects. Similarly small changes to constraints have small effects, if at all. More precisely, small changes to inactive and active constraints will have respectively no effect and linear effect on the optimal point [28]. **(c)** We could apply this analysis to several families of available compounds and test them on various normal and cancerous cell lines in cell culture assays. We are currently actively pursuing this approach.

Moreover, future investigators may be able to use animal models to examine the ratios of tubulin isotypes, both mRNA and protein, in a wide range of normal tissues. The advantage of a mouse model is that the tissue can be fresh enough to still preserve the mRNA. The protein levels can be checked in, for example, bovine or porcine tissues. We anticipate that the relative levels of the different isotypes are likely to be somewhat invariant among mammals, for any given tissue. Therefore if, for instance, liver has a certain ratio of isotypes in the mouse and the cow, then it is likely that humans do as well. In other words, if rodents and ungulates have the same ratios, primates probably do also. This strategy can be exploited in designing animal trials for drugs targeting specific types of cancers.

### Generality of this framework

While this paper deals with tubulin isotypes, this same mathematical framework can be applied to any other class of molecular target.

When multiple drugs from the same family of compounds have been derivatized to obtain different binding affinities with respect to the different β-tubulin isotypes, we may assume that they are non-interacting and combine to produce an additive effect on the cells. Here, this OTIP_sum_ model will allow us to easily consider a combination of drugs (*d*_1_, *d*_2_), in proportions (*α*, 1 - *α*) for any *α* ∈ [0,1], using the obvious “cumulative profile”,

(8) $$r\left(\alpha d_{1}\;+\;\left(1-\alpha \right)d_{2}\right)\;=\;\mathrm{\alpha r}\left(d_{1}\right)\;+\;\left(1-\alpha \right)r\left(d_{2}\right).$$

This means we can consider using a combination of drugs, taken in a proportion reflecting the affinities for several resultant tubulin targets, as a more efficacious treatment regimen that that offered by a single optimized drug entity. For example, instead of a drug with a profile of 50% βI, 25% βII and 25% βIII, we could instead use a combination of three drugs, each of which targets narrowly each of the three tubulin isoforms, and taken in the proportion 50%, 25%, and 25%, respectively.

## Conclusions

We can use this analysis as part of a rational drug design process, to predict the effects of a specific drug, based on its known binding affinities for each tubulin isotype. For example, many researchers have suggested that an effective cancer chemotherapy drug is one that binds most strongly to the βIII isotype, as βIII is found in a wide variety of cancers, but occurs infrequently in healthy cells [11,36]. This has motivated several groups to design drugs that target the βIII isotype, including novel derivatives of paclitaxel and colchicine [20], both currently in preclinical testing and development. Our OTIP framework extends this, by suggesting the *profile* for a drug that will bind strongly to the β-tubulin isotypes of a given cancer, but only weakly to those of healthy cells. This analysis can also use this analysis to help understand many other tubulin-targeting drug families that are now under active investigation – including laulimalide, peloruside A, noscapine, epothilones and discodermolide [1,39,40]. For example, recent binding energy calculations show that laulimalide preferentially binds to the βV isotype and peloruside A to the βI isotype [19]. While there is currently no detailed information about the binding energies of other drugs for tubulin isotypes, the analysis in this paper motivates the need for such determinations, as that binding information would allow us now to use OTIP’s formulation to quantitatively predict the effectiveness of these drugs, in terms of how much their β-tubulin isotype binding profile will damage a given cancer type, versus damaging important healthy cells. Finally, this method could be applied to other classes of biological targets, in addition to tubulin isotypes.

## Abbreviations

AC: Adenocarcinoma; ER: Estrogen receptor; IDC: Infiltrating ductal carcinoma; IV: Intravenous; LAU: Laulimalide; mRNA: Messenger ribonucleic acid; NSC: Non-small-cell; OTIP: Optimal tubulin isotype profile; PD: Poorly differentiated; PELA: Peloruside A; SC: Serous carcinoma; SCC: Squamous cell carcinoma; WD: Well differentiated.

## Competing interests

The authors declare that they have no competing interests.

## Authors’ contributions

JAT conceived the project. RG and SR developed a computational algorithm. SR carried out simulations. MG performed the peloruside and laulimalide binding calculations. All authors wrote the paper. All authors read and approved the final manuscript.
